# High-Temperature Growth of CeO_*x*_ on Au(111) and Behavior under Reducing and Oxidizing Conditions

**DOI:** 10.1021/acs.jpcc.4c08072

**Published:** 2025-02-12

**Authors:** Rudi Tschammer, Lars Buß, Emilia Pożarowska, Carlos Morales, Sanjaya D. Senanayake, Mauricio J. Prieto, Liviu C. Tănase, Lucas de Souza Caldas, Aarti Tiwari, Thomas Schmidt, Miguel A. Niño, Michael Foerster, Jens Falta, Jan Ingo Flege

**Affiliations:** † Applied Physics and Semiconductor Spectroscopy, Brandenburg University of Technology, 03046 Cottbus, Germany; ‡ Institute of Solid State Physics, University of Bremen, 28359 Bremen, Germany; § Chemistry Division, Brookhaven National Laboratory, Upton, New York 11973, United States; ∥ Department of Interface Science, Fritz-Haber-Institute of the Max-Planck Society, 14195 Berlin, Germany; ⊥ ALBA Synchrotron Light Facility, 08290 Cerdanyola del Vallès, Spain; # MAPEX Center for Materials and Processes, University of Bremen, 28359 Bremen, Germany

## Abstract

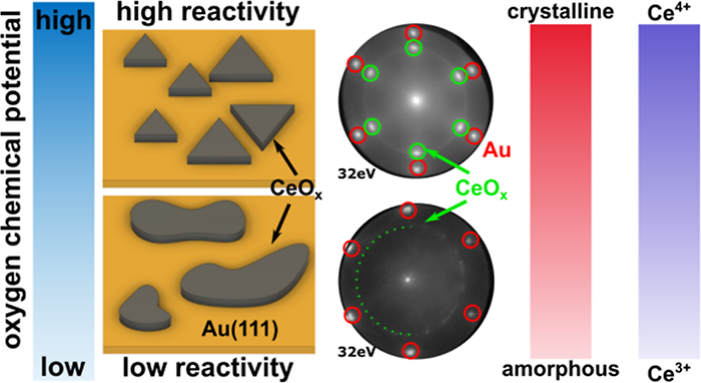

Inverse oxide–metal model catalysts can show superior activity and selectivity compared with the traditional supported metal–oxide architecture, commonly attributed to the synergistic overlayer–support interaction. We have investigated the growth and redox properties of ceria nanoislands grown on Au(111) between 700 and 890 °C, which yields the CeO_2_–Au(111) model catalyst system. We have observed a distinct correlation between deposition temperature, structural order, and oxide composition through low-energy electron microscopy, low-energy electron diffraction, intensity–voltage curves, and X-ray absorption spectroscopy. Improved structural order and thermal stability of the oxide have been achieved by increasing the oxygen chemical potential at the substrate surface using reactive oxygen (O/O_2_) instead of molecular O_2_ during growth. In situ characterization under reducing (H_2_) and oxidizing atmospheres (O_2_, CO_2_) indicates an irreversible loss of structural order and redox activity at high reduction temperatures, while moderate temperatures result in partial decomposition of the ceria nanoislands (Ce^3+^/Ce^4+^) to metallic cerium (Ce^0^). The weak interaction between Au(111) and CeO_*x*_ would facilitate its reduction to the Ce^0^ metallic state, especially considering the comparatively strong interaction between Ce^0^ and Au^0^. Besides, the higher reactivity of atomic oxygen promotes a stronger interaction between the gold and oxide islands during the nucleation process, explaining the improved stability. Thus, we propose that by driving the nucleation and growth of the ceria/Au system in a highly oxidizing regime, novel chemical properties can be obtained.

## 1 Introduction

As the consequences of progressing climate change caused by anthropogenic greenhouse gas emissions are becoming ever more apparent, there is a pressing need to limit and sequester carbon emissions.^1^ In the short term, the efficient use of fossil-fuel-derived feedstocks in the chemical industry and, in the long term, the stepwise transformation toward renewable feedstocks and circular production carbon cycles are of paramount importance.^2^ This new paradigm necessitates the development of new and improved catalysts for existing and emerging production chains, e.g., converting CO_2_ into fuels like methanol.^3,4^ To understand the physicochemical behavior and potential optimization routes of the many different and complex catalytic systems used in industrial applications,^5^ surface-science-based studies have been carried out on several model systems in the last decades, traditionally consisting of metal nanoparticles dispersed on oxide substrates.^6−8^

Contrary to this approach, Rodriguez et al. showed that so-called inverse metal–oxide catalysts, consisting of oxide particles dispersed on a metal support, can exhibit superior catalytic activity compared to their metal-on-oxide counterparts.^9,10^ Particularly for CO_2_ conversion into methanol, the CeO_*x*_/Cu(111) system has been shown to perform considerably better than its counterpart Cu/CeO_*x*_ and traditional Cu–Zn catalysts.^11,12^ The basic functionality of this catalyst architecture comprises a reducible oxide capable of binding the CO_2_ molecule in an activated form together with a metal support, which provides atomic hydrogen for the reaction by dissociating molecular H_2_. The promising behavior of inverse metal–oxide catalysts has been attributed to the unique interfacial sites resulting from a synergistic interaction between the constituents combined with the increased number of defect sites on the oxide nanoparticle.^13,14^ In this context, ceria represents an outstanding candidate for multiple catalytic applications thanks to the reversible release and storage of oxygen by transitioning from higher to lower oxidation states, i.e., Ce^4+^ to Ce^3+^ and vice versa.^15−17^ The interaction of ceria with the metallic support has been studied on a variety of model systems, including Au(111),^18,19^ Cu(111),^20^ Ru(0001),^21^ and Pt(111),^22,23^ gaining valuable insights on the redox behavior of ceria and the influence of the metal substrate on stability and activity.

In this context, the inert Au(111) surface offers an ideal platform for studying the ceria component isolated from more active metallic supports, simplifying this complex interaction as gold shows only mild interaction with oxide particles.^24^ Surprisingly, the deposition of both cerium metal^24^ and cerium oxide^19,25,26^ onto the Au(111) surface has been the subject of a limited number of publications, regardless of its advantages, compared to catalytically more active^27^ substrates such as Cu(111) (e.g.,^28,29^) and Ru(0001) (e.g.,^21,30,31^). Therefore, we have aimed to investigate further the constituents of the CeO_2_–Au systems and their constitutional interplay, using advanced surface in situ structure-sensitive and real-time measurements while relying on a careful modulation of the metal–oxide component and thus elucidating their interactions.

Due to the unique Au(111) herringbone reconstruction,^32^ its surface exhibits a periodic arrangement of different adsorption sites, significantly determining the nucleation and morphology of ceria nanoparticles.^24^ For example, the anisotropic diffusion resulting from the presence of the surface reconstruction can lead to the formation of elongated oxide particles,^19^ while bilayer islands seem to be the most stable configuration when growing at moderate temperatures.^26^ Besides, gold only has a weak interaction with cerium oxide, compared to a relatively strong interaction with cerium metal and a tendency toward alloy formation.^24^ Other studies have focused on exploring the catalytic activity of CeO_*x*_/Au(111),^9^ investigating the reaction pathway of the water–gas shift reaction (WGSR),^33^ CO oxidation,^34^ and ethanol decomposition.^35^ To date, there are no studies on the high-temperature growth of ceria on Au(111) above 530 °C to determine the influence of the growth temperature on the structural and chemical properties when providing sufficient thermal energy to overcome kinetic barriers.

This work presents the deposition of ceria by reactive thermal evaporation of metallic cerium under oxidizing (O_2_ and O/O_2_) atmospheres at elevated temperatures between 700 and 890 °C. The as-grown surfaces have been first characterized by low-energy electron microscopy (LEEM), low-energy electron diffraction (LEED), and intensity–voltage LEEM (I(V)–LEEM) as well as X-ray absorption spectroscopy (XAS). The system has subsequently been exposed to different reducing and oxidizing environments, following the structural and chemical changes in situ and in real-time. Using the same experimental tool kit, we have observed a distinct relationship between the crystallinity of the as-grown oxide particles and the deposition temperature as well as a correlation between crystallinity and redox behavior. The comparison of these results with those obtained from thoroughly characterized systems such as ceria on Cu(111) and Ru(0001) illustrates the role of the complex interaction between ceria and metallic supports, helping in the design of the next generation of highly efficient catalysts.

## 2 Experimental Methods

A commercially available polished Au(111) single crystal (Mateck) with a nominal miscut of less than 0.1° was used as a substrate. A clean crystal surface was achieved by repeated cycles of sputtering with Ar^+^ ions (5 × 10^–5^ mbar) followed by subsequent annealing to temperatures between 600 and 1000 °C. Surface quality was asserted by the presence of well-defined spots of the herringbone reconstruction in LEED, crisp and smooth LEEM images, and characteristic I(V)–LEEM curves. The same procedure was also used to restore a clean surface for subsequent experiments.

Ceria deposition was achieved by reactive thermal evaporation of cerium metal (Alfa Aesar, 99.9%) from a molybdenum crucible mounted in a Focus EFM 3 electron beam evaporator, typically in an oxygen ambient of 5 × 10^–7^ mbar unless otherwise noted, similar to established recipes.^21,30,31^ Substrate temperature has been varied between 700 and 890 °C using the electron beam heating of LEEM III sample holders and measured by the built-in thermocouple. In experiments carried out at the University of Bremen, reactive oxygen (10–15% O in O_2_) has been provided by an oxygen cracker from Dr. Eberl MBE-Komponenten GmbH. The cracking rate has been estimated by using chamber pressure and pump rate to convert oxygen pressure to flow rate and comparing with the specifications provided by the manufacturer.

The measurements described herein have been carried out with three different LEEM instruments:

- A commercial Elmitec LEEM III instrument equipped with an energy analyzer providing a spatial resolution of approximately 10 nm at the University of Bremen.
- The LEEM/PEEM endstation of the CIRCE beamline of the ALBA synchrotron based on a commercial Elmitec LEEM III instrument with a spatial resolution of 10 nm in LEEM/PEEM mode and 20 nm in XPEEM measurements.^36^
- The SMART spectro-microscope at the UE49PGM beamline of the BESSY II synchrotron operated by the Helmholtz Center Berlin, offering a spatial resolution of 2.6 nm in LEEM, 10 nm in PEEM, and 18 nm in XPEEM.^37^

All microscopes operate with a base pressure of 1 × 10^–10^ mbar and can be equipped with evaporators and leak valves, allowing for in situ sample preparation and measurements in gas pressures up to 1 × 10^–5^ mbar. The instruments at the University of Bremen and ALBA synchrotron facility use a LaB_6_ crystal as an electron source and are equipped with hemispherical image analyzers, while the SMART instrument utilizes a field-emission gun in the imaging column and a custom-designed energy analyzer. XAS spectra of the Ce M_4,5_-edge have been recorded using secondary electron yield scanning from 870 to 910 eV, although only the Ce M_5_-edge is shown here. μLEED images could be recorded from areas as small as 250 nm in diameter by using different apertures.

The oxidation state of the cerium cations has been derived by fitting a linear combination of reference XAS spectra of CeO_2_/Ru(0001) and Ce_2_O_3_/Ru(0001) recorded at other spectroscopic photoemission and low-energy electron microscope instruments as part of previous studies.^38^ An analogous procedure has been adopted for the analysis of the I(V)–LEEM curves of the different growths, using reference curves that are representative of clean Au(111), CeO_2_, and Ce_2_O_3_ from CeO_2_/Ru(0001) and Ce_2_O_3_/Ru(0001),^38,39^ respectively; the reference for Ce metal was extracted from supplementary Ce/Au(111) experiments. This kind of fitting methodology^40^ consisting of a linear combination of I(V) reference curves has already been used successfully for other systems.^39,41^

Particle size and coverage were estimated from LEEM images of as-deposited samples using the built-in “Analyze Particles” function of the ImageJ software.^42^ The threshold value was manually adjusted until proper discrimination between the individual particles was achieved.

## 3 Results and Discussion

### 3.1 Influence of Substrate Temperature on CeO_*x*_/Au(111) Growth

This section describes the deposition of cerium oxide on clean Au(111) between 700 and 890 °C by evaporating metallic cerium in an O_2_ atmosphere of 5 × 10^–7^ mbar, mainly focusing on the structural properties and the oxidation state of the oxide particles as a function of deposition temperature.

**Figure 1:**
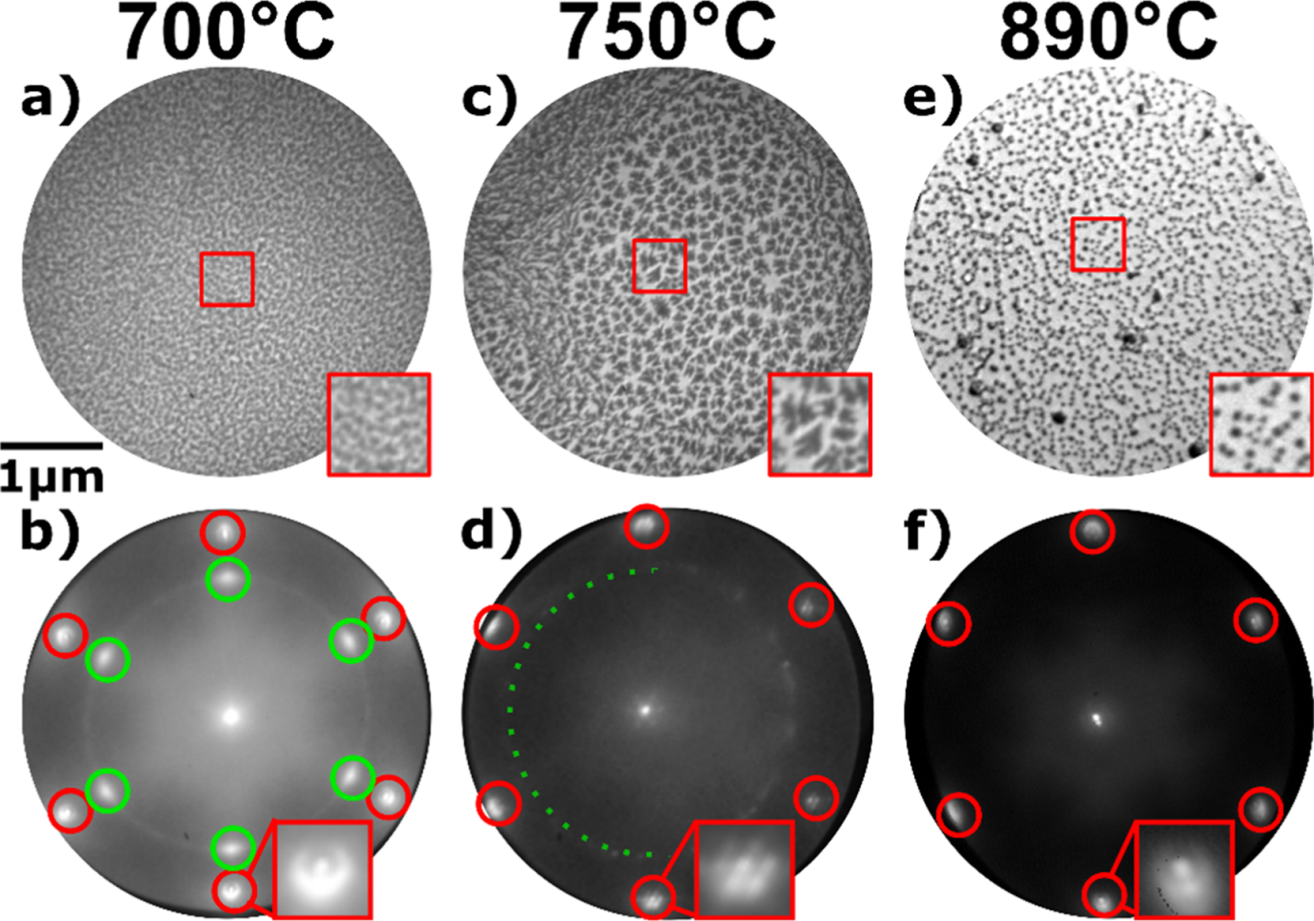
LEEM images and corresponding μ-LEED patterns of CeO_*x*_/Au(111) grown under an O_2_ atmosphere at 700 °C (a, 15 eV; b, 32 eV), 750 °C (c, 15 eV; d 32 eV), and 890 °C (e, 18 eV; f, 32 eV), respectively. Insets of the LEEM images show a close-up of the respective highlighted region with a side length of 500 nm. In LEED patterns, red circles mark the Au(111) spots, green circles indicate the cerium oxide contribution, and red squares show a close-up of a Au(111) spot.

Figure 1 shows LEEM images and the corresponding μ-LEED patterns of CeO_*x*_ grown on Au(111) at different temperatures. Figure 1a shows cerium oxide deposited at 700 °C, resulting in tiny, dark oxide nanoparticles that can hardly be resolved individually. The average particle size has been determined to be approximately 38 nm at a fractional coverage of 0.18; previous AFM measurements (not shown) indicated the island height to be on the order of a few nanometers. The corresponding LEED pattern (Figure 1b) confirms the presence of clean Au(111) (red circles), including the well-known herringbone reconstruction,^32,43,44^ and CeO_*x*_(111) islands in azimuthal registry with the substrate (green circles), as well as randomly oriented ceria islands (faint ring corresponding to an identical in-plane lattice constant of random azimuthal orientation). Increasing the deposition temperature to 750 °C leads to an increase in nanoparticle size to approximately 72 nm at a fractional coverage of 0.28, as observed in Figure 1c. Concomitantly, the island density is decreased, which has also been observed for CeO_*x*_ grown on other metal surfaces.^30,41,45^ However, the as-grown ceria islands do not exhibit the well-defined triangular shape observed for other systems, such as (111)-oriented CeO_2_ on Ru(0001)^30^ and Cu(111).^46^ In addition, the LEED pattern (Figure 1d) shows an absence of the intense ceria spots mentioned previously, instead only displaying a faint, inhomogeneous ring (green, dotted half-circle), indicating the growth of ceria nanoparticles composed of small crystallites of almost random azimuthal orientation. This polycrystalline nature can also be regarded as the reason for the jagged appearance of the nanoparticle outline and may constitute a potential explanation for the increased nanoparticle size. The difference in the features attributed to the herringbone reconstruction around the first-order Au spots in Figure 1b,d is related to the different acquisition temperatures and reflects the change in surface reconstruction described by Abernathy and co-workers.^47^

Increasing the temperature further to 890 °C during the growth process results in smaller, dark oxide particles with an average size of 44 nm and a coverage of 0.17 (Figure 1e), in stark contrast to the evolution of island size with deposition temperature observed for CeO_2_(111)/Ru(0001)^30^ and CeO_2_(111)/Cu(111).^46^ While no conclusive reason for the decrease in island size can be derived from the measurements presented here, a potential explanation might be based on a reduction of the oxidation state of the cerium oxide precursor species, possibly increasing the tendency for alloying with the substrate and hence a decreased diffusion length on the Au(111) surface. This scenario would favor the nucleation of small islands at special sites or local surface defects of the herringbone reconstruction, distorted by substrate temperature, over extended diffusion and attachment to existing nanoislands. Interestingly, the sizes of the CeO_*x*_ nanoislands observed in the experiments described in this section differ strongly from those observed on more reactive substrates such as Ru(0001)^30^ and Cu(111),^46^ typically ranging from 100 nm to several microns, while exhibiting certain similarities in island size to CeO_*x*_ nanoparticles deposited on Pt(111),^41^ a less oxygen-affine substrate. The low oxygen potential at the Au(111) surface would hinder the formation of large, well-ordered, and stoichiometric CeO_2_ islands due to the low mobility of the cerium oxide precursor species, as mentioned previously.

**Figure 2:**
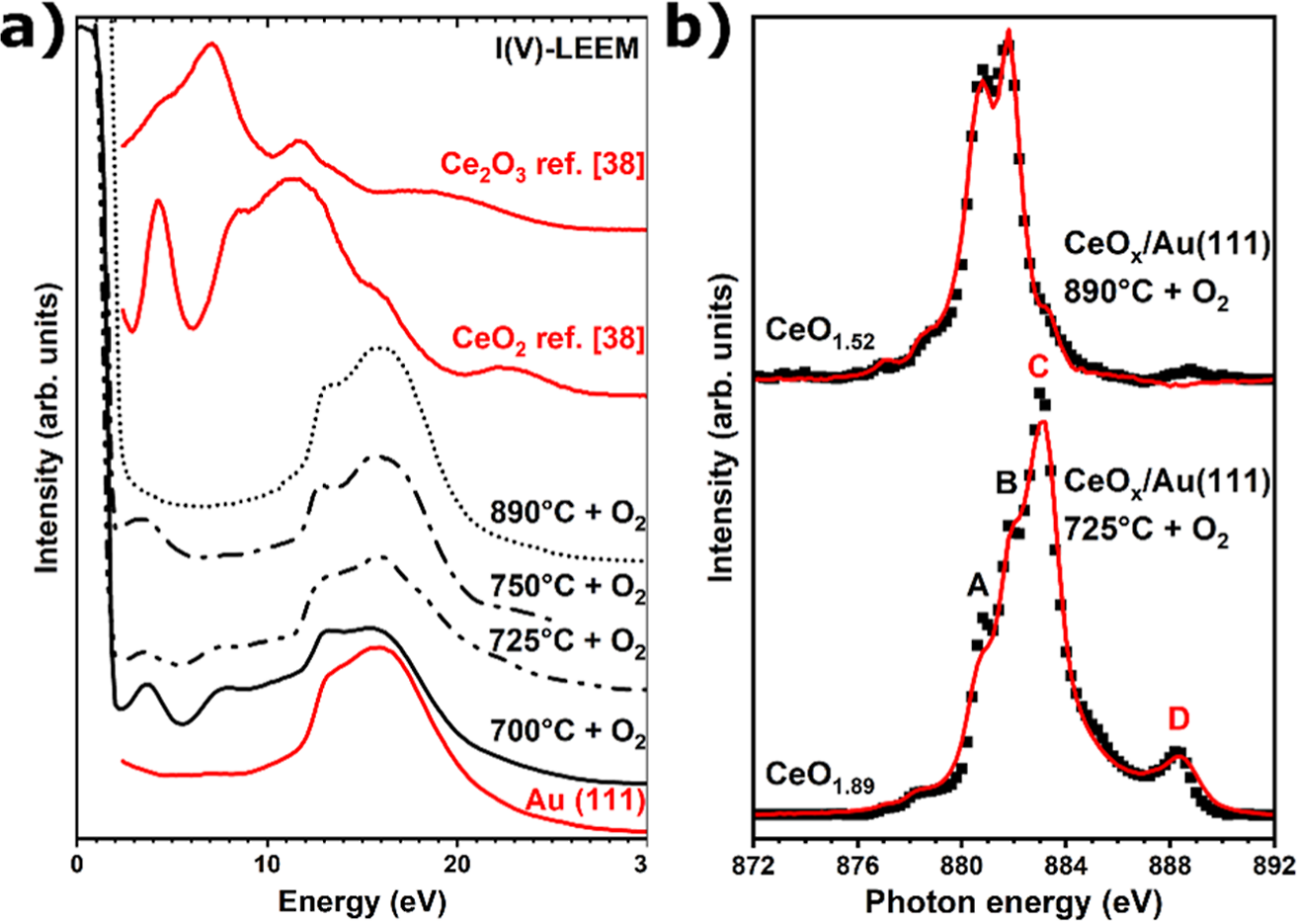
(a) I(V)–LEEM curves of cerium oxide grown under an O_2_ atmosphere at 700, 725, 750, and 890 °C (black curves) and reference curves for Au(111), Ce_2_O_3_(111), and CeO_2_(111) (red curves),^38,39^ (b) Ce M_5_-edge XAS spectra of cerium oxide/Au(111) grown at 725 °C and at 890 °C with O_2_ (black dots) and fit using reference curves (red line).

The absence of any additional spots compared to those from Au(111) in the acquired LEED image (Figure 1f) indicates the formation of fully amorphous CeO_*x*_ particles at high temperatures. The presence of cerium oxide on the sample is confirmed by XAS measurements of the Ce M_5_-edge (see Figure 2b).

The presence of cerium oxide is also evident from additional peaks in the I(V)–LEEM curves of the different growths shown in Figure 2a. In more detail, the I(V)–LEEM analysis offers, by comparing the experimental curves with those obtained from reference structures, a fingerprint to identify the cerium local atomic structure of well-ordered deposits and associated oxidation.^48^ The I(V)–LEEM curve of clean Au(111) as well as CeO_2_(111)/Ru(0001) and Ce_2_O_3_(111)/Ru(0001) has been included for direct comparison (Figure 2a, red curves).^38,39^ Deposition at 700 °C results in additional features at electron energies around 3–4 and 7–12 eV, similar to those expected for low coverages of CeO_2_ as deducted from the corresponding reference curve. A slight increase in deposition temperature to 725 °C results in a very similar I(V) curve, but with lower intensity, a trend that continues at 750 °C, where the feature around 7–12 eV vanishes, and that is completed at around 890 °C where the I(V)–LEEM curve only shows the Au(111) features. Similar to the LEED results, this evolution can be explained by an apparent loss of crystallinity of the oxide nanoislands, implying that the peak around 3–4 eV originates from the nanocrystallites formed up to 750 °C.

Figure 2b depicts the Ce M_5_-edge XAS spectra of the CeO_*x*_/Au(111) growth at 725 and 890 °C, showing a clear difference between the deposition at moderate (725 °C) and high (890 °C) temperatures. The presence of four distinct peaks in the XAS spectrum of ceria grown at 725 °C implies a mixture of Ce^3+^ (A, B) and Ce^4+^ (C, D) cations with a predominance of the latter, while the two-peak structure of the high-temperature deposition reflects the dominance of the Ce^3+^ oxidation state.^49,50^ Fitting both spectra by a simple linear combination of Ce^3+^ and Ce^4+^ reference spectra yields a composition of CeO_1.89_ and CeO_1.52_ for CeO_*x*_/Au(111) deposited at moderate and high temperatures, respectively.

The measurements presented up to now show a clear correlation between the growth temperature and the structural order of the as-grown ceria nanoparticles, as inferred from the corresponding μ-LEED and I(V)–LEEM analyses. Also, the observed trend for nucleation density and island size clearly differs from a simple Arrhenius-like behavior observed for CeO_2_/Ru(0001),^30^ i.e., a continuous decrease in island density and increase in island size with temperature. A more detailed discussion of the influence of the substrate–oxygen interaction on the evolution of island size with increasing deposition temperature is relegated to the Discussion section. Furthermore, the cation oxidation state changes from Ce^4+^ to Ce^3+^ when the deposition temperature is increased from 700 to 890 °C.

A possible explanation for the observed nonmonotonic relationship between island size and deposition temperature and the generally small island size compared to other substrates might be the particularly weak interaction between Au and oxygen. As shown for other substrates,^30,41,46^ the presence of adsorbed oxygen can be identified by a characteristic superstructure observed in μLEED-patterns, which may prime the surface and favor a layer-by-layer growth mode over island growth, yielding predominant growth in the lateral direction and, therefore, larger islands. Au(111) seems to differ from this behavior, as no superstructure attributed to oxygen is identified during the experiments presented here, which could promote the formation of smaller, three-dimensional nanoparticles over large, quasi-two-dimensional deposits.

Additionally, the lack of a distinct oxygen–substrate interaction may result in increased prominence of other factors, such as the herringbone reconstruction of the Au(111) surface providing special adsorption sites^19^ and the anisotropic diffusion parallel to the reconstruction versus across it.^25^ As the herringbone reconstruction experiences changes with substrate temperature as evidenced by changes in the μLEED patterns presented in this section and reported by Abernathy and co-workers,^47^ resulting in potential changes to adsorption sites attributed to the reconstruction, and diffusion is generally understood to be a thermally activated process, these factors may explain the nonmonotonic evolution of island size as a function of temperature.

To summarize, the increased particle size of CeO_*x*_ nanoislands deposited at 750 °C in O_2_ ambient may be caused by the enhanced diffusion of nanocrystallites across the Au(111) surface, attaching to existing nanoislands nucleated on surface defects, e.g., provided by the herringbone reconstruction. Increasing the temperature further to 890 °C may lead to a decrease in particle size due to the preferential nucleation of CeO_*x*_ precursor species at surface defects, combined with lower mobility on the surface, and island growth over the attachment of CeO_*x*_ precursors to existing islands due to the weak gold–oxygen interaction.

### 3.2 Influence of Oxygen Chemical Potential on CeO_*x*_/Au(111) Growth

The influence of the oxygen chemical potential on the growth of ceria/Au(111) has been investigated at a deposition temperature of 750 °C using an atomic oxygen source to provide a mixture of atomic oxygen and molecular oxygen (10–15% O in O_2_), henceforth referred to as reactive oxygen (O/O_2_). The selection of this growth temperature is explained by being the highest temperature at which ceria retains some ordered structure, i.e., azimuthally randomly oriented nanocrystals. Thus, if the oxygen chemical potential affects the ceria growth on Au(111) in terms of morphology and structural order, as has been shown for other systems, it will be readily apparent in this temperature range.

**Figure 3:**
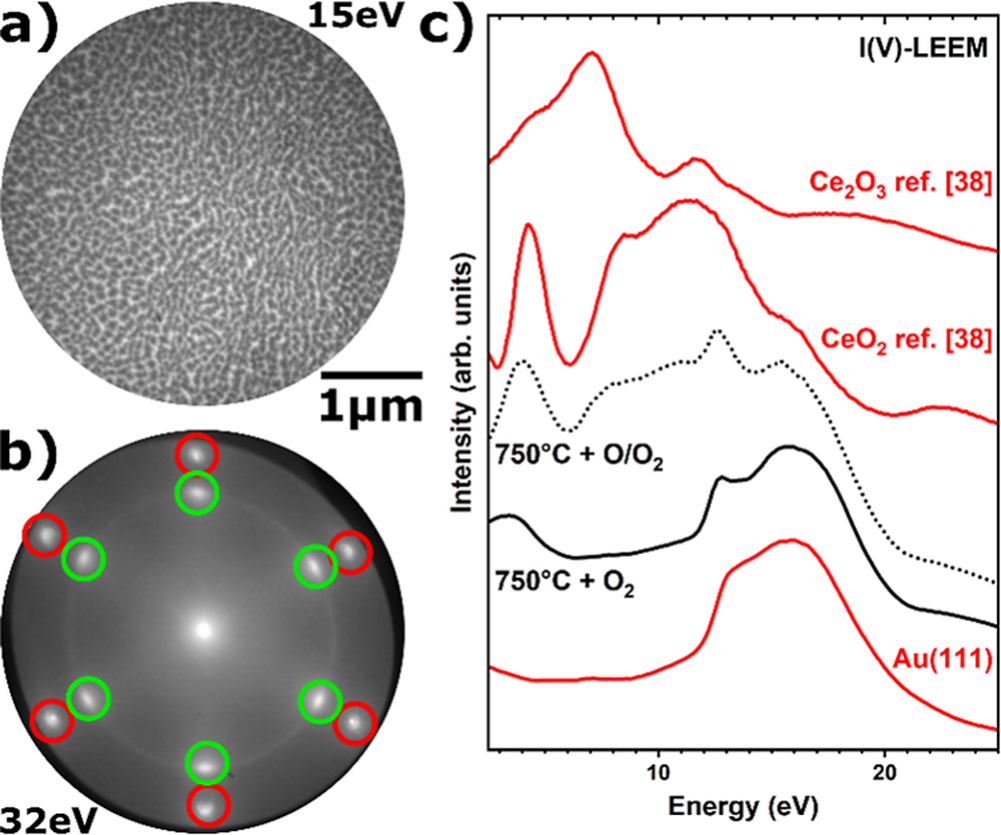
(a) LEEM and (b) μ-LEED pattern of CeO_*x*_/Au(111) grown at 750 °C with O/O_2_. Red and green circles in the μ-LEED pattern indicate gold and ceria spots, respectively. (c) Comparison of I(V)–LEEM curves for ceria on Au(111) deposited at 750 °C in O_2_ (black line) and O/O_2_ (dotted line). I(V)–LEEM reference curves for Au(111), CeO_2_, and Ce_2_O_3_ are shown as red lines.^38,39^

Comparing the LEEM and LEED images of the growths with molecular oxygen (O_2_) and reactive oxygen (O/O_2_) (Figures 1c,d and 3a,b, respectively), the use of O/O_2_ results in smaller islands at higher nucleation density than when using O_2_, thus exhibiting features similar to CeO_*x*_/Au(111) grown at lower temperatures (see Figure 1a,b). The average particle size of the sample prepared with reactive oxygen was determined to be 56 nm at a fractional coverage of 0.24. However, the particle size estimation for this sample is skewed toward larger particle sizes due to the close proximity of the nanoislands, leading to difficulties in proper differentiation during particle identification. In the corresponding μ-LEED pattern shown in Figure 3b, intense spots (green circles) emerge, indicating the growth of well-ordered CeO_*x*_ (111)-oriented islands in registry with Au(111), combined with a faint ring of ceria(111) with random azimuthal orientation. Furthermore, the comparison of the I(V)–LEEM curves of ceria grown at 750 °C using these two different oxygen sources (Figure 3c) confirms the increased structural order resulting from the use of O/O_2_, with distinct features appearing in the electron energy ranges of 3–4 and 7–12 eV that recall the reference curve of CeO_2_.

This increase in structural order is likely due to a stronger interaction of the atomic oxygen from the O/O_2_ mixture with the Au(111) surface, resulting in enhanced nucleation and adhesion of small ceria clusters. Similar variations in the growth mode have been reported in other systems, such as Cu(111), where the increase of the adsorbed oxygen at the metallic surface also determines the nucleation and facet growth of ceria islands.^46^ Whereas the excess of oxygen on the Cu(111) surface resulted in the eventual suppression of CeO_2_ (111)-oriented islands in favor of (100)-oriented CeO_2_ and a much lower nucleation density due to the increased mobility of Ce and O species, the growth of ceria on Au(111) does not follow this trend, possibly due to the much lower affinity between oxygen and the inert Au(111) surface. The lower affinity for oxygen could directly explain the lower oxygen concentration at the surface, which results in a lower oxygen concentration in the growing cerium oxide nanoparticles, which is reflected by the lower average oxidation state of the Ce cations. Interestingly, as the higher oxidation state appears to correlate with increased structural order in the CeO_*x*_ particles, the presence of more oxygen on the gold surface likely increases the interfacial interaction necessary for enhanced ordering in the CeO_*x*_ deposit.

### 3.3 Temperature Stability of CeO_*x*_/Au(111)

After characterizing the as-grown CeO_*x*_/Au(111) systems, the stability of ceria deposited at 700 °C using O_2_ and of ceria prepared at 750 °C with O/O_2_ has been studied using LEED. Both samples have been kept at 415 °C in ultrahigh-vacuum (UHV) conditions (pressure ∼10^–9^ mbar) for 50 min (for 700 °C + molecular oxygen sample) and 10 h (750 °C + reactive oxygen sample), respectively, tracking possible changes in the LEED patterns.

**Figure 4:**
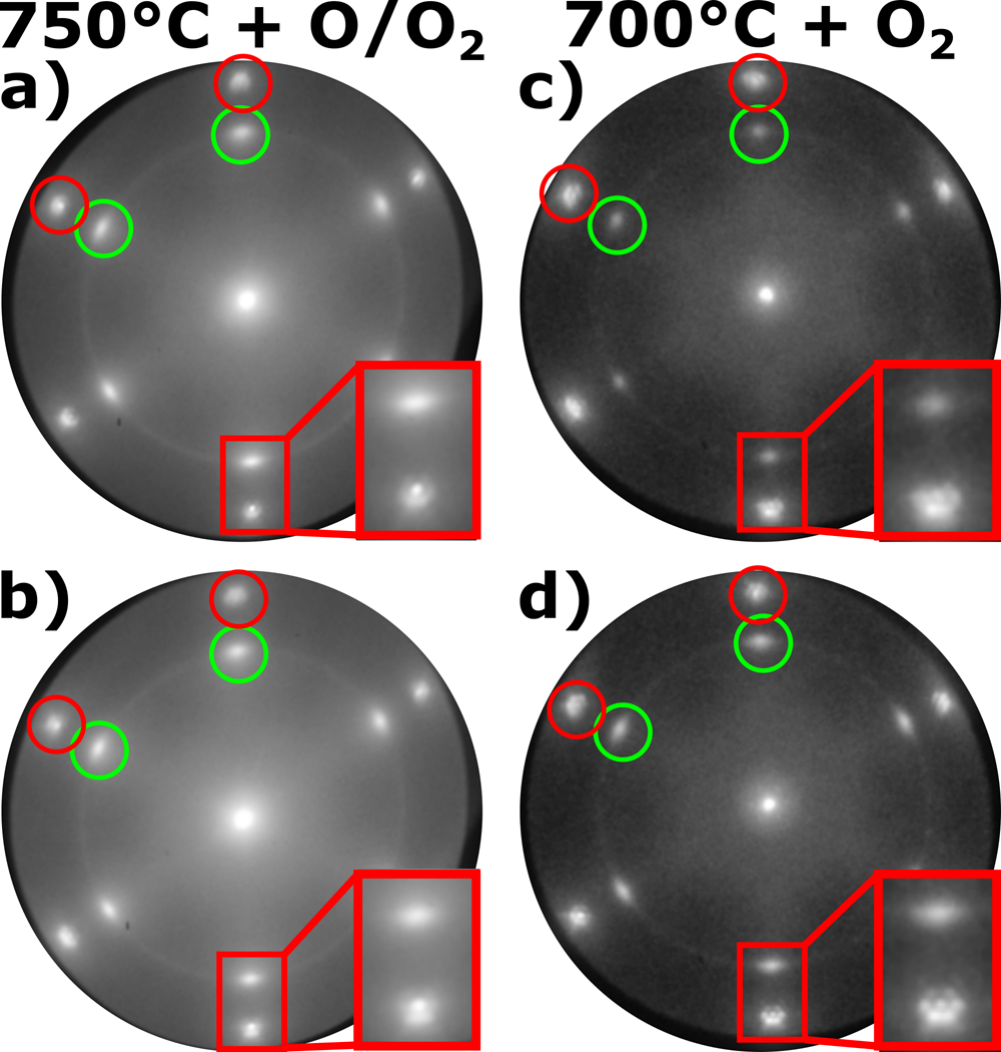
LEED images (electron energy 32 eV) of cerium oxide on Au(111) grown at 750 °C with reactive oxygen (a) as-deposited and (b) after 10 h at 415 °C in UHV as well as grown at 700 °C under an O_2_ atmosphere (c) after 50 min in UHV at 415 °C and (d) after reoxidation at 600 °C using O_2_.

The unique herringbone reconstruction of the Au(111) surface is evident in LEED patterns of clean Au(111) surfaces taken at 415 °C, appearing as additional spots around the first-order gold beams and the central (00)-beam (see as examples the insets in Figure 4b,d). In comparison, the long-range order of the reconstruction diminishes around 600 °C,^47^ resulting in halo-like features around the first-order gold spots (see as an example Figure 4a). LEED measurements of as-deposited CeO_*x*_/Au(111) deposited at 750 °C using O/O_2_ (Figure 4a) and after 10 h at 415 °C in UHV demonstrate that the ceria spots and the corresponding faint ring remain unaltered, indicating no adverse effects of this annealing temperature on the oxide nanoparticles. Moreover, the observed changes in the features associated with the herringbone reconstruction are caused by the different temperatures during LEED measurement, as previously indicated.

In contrast, an equivalent experiment performed on samples grown at 700 °C using O_2_ shows a blurring of the otherwise sharp herringbone features around the first-order gold spots in the LEED patterns, which could imply an interaction of some material with the pristine Au(111) surface, leading to a slow disappearance of the herringbone reconstruction after sufficient time. We note that the time needed for this change is below 50 min, while the previous sample grown under reactive oxygen remained unaltered for ten hours under equivalent conditions. However, the well-defined herringbone features can be restored by exposing the sample to molecular oxygen at temperatures above 550–600 °C, as shown in Figure 4d. Further implications of this observation and a possible explanation are discussed in the following section.

**Figure 5:**
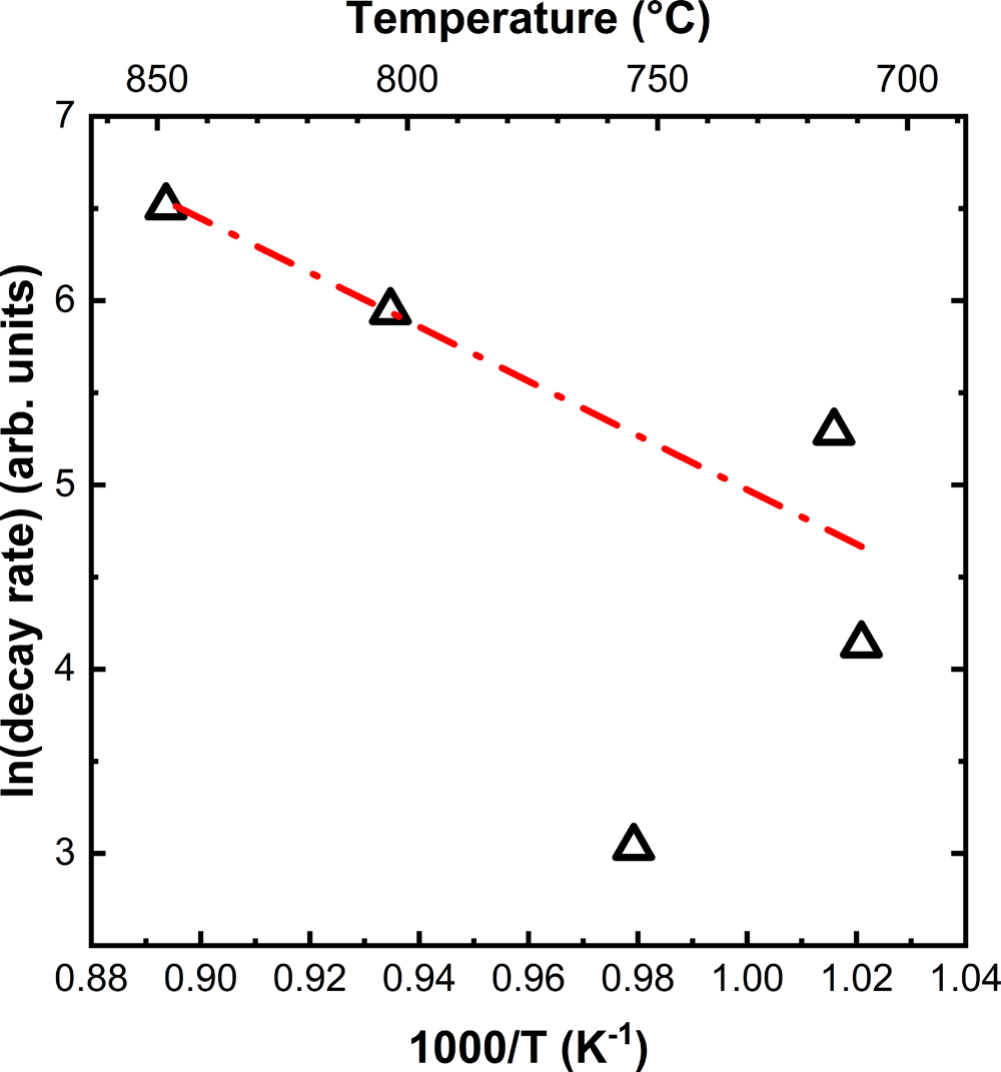
Arrhenius plot of the decay rate of cerium oxide spots in μLEED patterns of CeO_*x*_/Au(111) grown at 700 °C using O_2_, held at different temperatures (black triangles), and a linear fit (red dashed–dotted line), excluding the outlier at 750 °C.

The stability of well-ordered CeO_*x*_(111)/Au(111) grown at 700 °C using O_2_ has also been investigated by heating the sample to temperatures between 750 and 850 °C under an O_2_ pressure of 5 × 10^–7^ mbar while monitoring the intensity of the ceria spots in LEED. Plotting the intensity decay rate versus inverse temperature in the format of a typical Arrhenius plot (Figure 5) shows a noticeably lower decay rate around 750 °C, which might be related to a transformation from single-crystalline to polycrystalline ceria islands as observed when comparing CeO_*x*_/Au(111) grown at 700 °C in O_2_ and 750 °C in O_2_, as shown in Figure 1a–d. Fitting a linear function to the data excluding the mentioned outlier at 750 °C, an activation energy (*E*_act_) of about ∼1.3 eV can be extracted, likely corresponding to the energy associated with the transition from well-ordered CeO_*x*_(111) to partially or fully amorphous ceria particles when using molecular oxygen during the growth, resulting in the observed decrease in intensity of the ceria spots in LEED.

### 3.4 Behavior of CeO_*x*_/Au(111) during Exposure to Different Gases

Following the initial characterization, ceria grown under different conditions has been exposed to reducing (H_2_) and oxidizing (O_2_, CO_2_) gases at 5 × 10^–7^ mbar to study the redox behavior and the influence of structural properties resulting from the specific choice of growth conditions. During gas exposure, changes were monitored by real-time μ-LEED measurements, with subsequent characterization by LEEM, μ-LEED, I(V)–LEEM, and XAS at specific stages of the reduction/oxidation process. Gas exposure was stopped once no further changes were observed in the μ-LEED patterns for a period of time, typically around 5 min.

**Figure 6:**
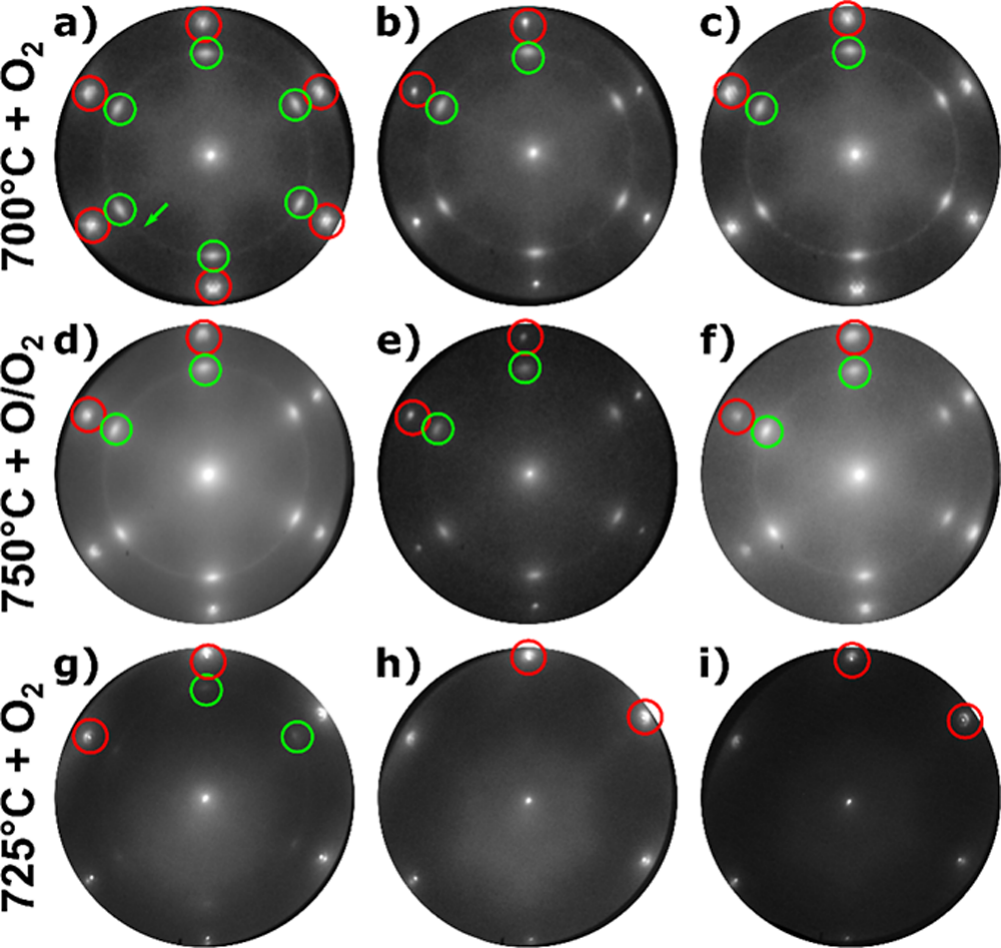
LEED images of CeO_*x*_/Au(111) grown at 700 °C with O_2_ (a) after postoxidation (32 eV), (b) after H_2_ reduction at 415 °C (32 eV), and (c) after reoxidation at 600 °C (32 eV) with O_2_; CeO_*x*_/Au(111) grown at 750 °C with O/O_2_ (d) as-grown (32 eV), (e) after H_2_ reduction at 415 °C (32 eV), and (f) after reoxidation with CO_2_ at 600 °C (32 eV); CeO_*x*_/Au(111) grown at 725 °C with O_2_ (g) after postoxidation (40 eV), (h) after H_2_ reduction at 600 °C (36 eV), and (i) after reoxidation with O_2_ at 700 °C (40 eV). Red and green circles indicate the gold and ceria contributions, respectively; the green arrow marks the faint ring caused by CeO_*x*_ nanoislands. Contrast has been adjusted for better visibility in images (g–i).

Figure 6 shows a collection of μ-LEED patterns of CeO_*x*_/Au(111) grown at 700 °C with O_2_ (Figure 6a–c), deposited at 750 °C using O/O_2_ (Figure 6d–f), and prepared at 725 °C with O_2_ (Figure 6g–i).

First, the postoxidized 700 °C growth (Figure 6a) was exposed to H_2_ at 415 °C for a total dose of 1.4 kL, resulting in the LEED pattern shown in Figure 6b. Compared to the LEED pattern of the as-grown ceria, a distinct change can be observed in the features around the first-order Au spots, corresponding to a complete disappearance of the herringbone reconstruction, as no additional features can be seen around the gold spots. This peculiar behavior appears to represent the next step following the blurring of the herringbone features mentioned in the previous section, indicating changes to the formerly pristine Au(111) surface. At the same time, the ceria spots experience an increase in intensity and a noticeable azimuthal broadening but no significant shift in the radial distance from the (00) beam that would indicate a change in the in-plane lattice parameter. However, these changes are fully reversed by reoxidizing the sample at 600 °C using an O_2_ dose of 5.4 kL.

Furthermore, the sample prepared at 750 °C under an O/O_2_ environment follows a similar evolution when exposed to H_2_ (dose of 1.8 kL), exhibiting the changes mentioned above to the gold and ceria spots, while the in-plane lattice constant of the oxide remains unaffected. Instead of annealing the sample in oxygen, CO_2_ at 5 × 10^–7^ mbar was used (6.2 kL dose), yielding the LEED pattern in Figure 6f. While most of the changes observed during reduction are reversed after dosing CO_2_, the features of the herringbone reconstruction remain halo-like instead of the sharp spots expected for a well-ordered Au(111) surface and as observed after postoxidation with O_2_, indicating an incomplete reversal of the changes to the gold substrate, likely due to the weaker oxidative capabilities of CO_2_ compared to that of O_2_. Contrary to these observations, reduction attempts of amorphous ceria nanoparticles prepared at 890 °C in an O_2_ atmosphere show no change in the herringbone reconstruction in μ-LEED patterns, dismissing a possible destabilization of the herringbone reconstruction on the pristine Au(111) substrate under reducing conditions.

Finally, by increasing the temperature during the reduction step to 600 °C (*p*_H_2__ = 5 × 10^–7^ mbar, 375 L) for the sample deposited at 725 °C using O_2_, the weak ceria spots of the as-grown μ-LEED pattern (Figure 6g) practically vanished, consistent with the disappearance of the CeO_*x*_-related features in the I(V)–LEEM curve (not shown). Interestingly, postoxidation at 700 °C using O_2_ (dose of 650 L) does not yield any recovery of ceria contributions in LEED (Figure 6i), implying that the loss of structural order during the reduction step is a nonreversible process.

**Figure 7:**
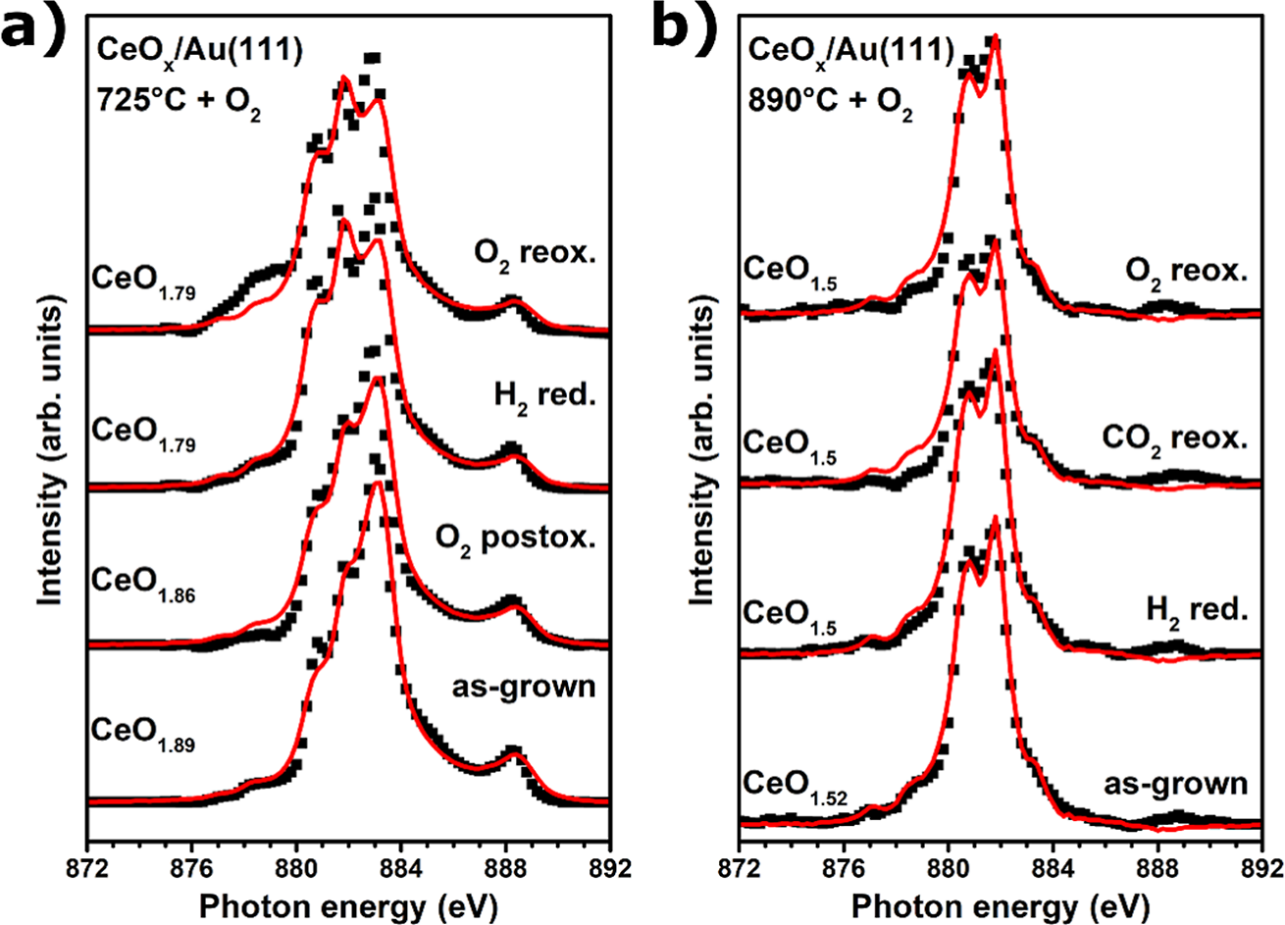
Ce M_5_-edge XAS spectra of (a, from bottom to top) CeO_*x*_/Au(111) grown at 725 °C with O_2_ as-grown, after O_2_ postoxidation at 600 °C, H_2_ reduction at 600 °C, and O_2_ reoxidation at 700 °C (black spots); (b, from bottom to top) as-grown CeO_*x*_/Au(111) grown at 890 °C with O_2_, after H_2_ reduction at 415 °C, CO_2_ reoxidation at 600 °C, and O_2_ reoxidation at 600 °C (black spots). The fit of XAS spectra using reference CeO_2_ and Ce_2_O_3_ spectra is depicted by red lines.

Further characterization was conducted using the XAS spectra acquired at the Ce M_5_-edge during different stages of the sample treatment. The oxidation states were derived by fitting the measured spectra with a linear combination of reference spectra for CeO_2_ and Ce_2_O_3_. The measurements and fits obtained for ceria/Au(111) prepared at 725 °C under an O_2_ atmosphere are shown in Figure 7a. The as-grown sample appears slightly reduced with a composition of CeO_1.89_, in line with the previously described relationship between the ceria oxidation state and growth temperature. Postoxidation with O_2_ at 700 °C (*p*_O_2__ = 5 × 10^–7^ mbar, 500 L dose) yields no increase in oxidation state, whereas the aforementioned high-temperature reduction at 600 °C leads to a significant reduction to CeO_1.79_, which cannot be reversed by subsequent oxygen reoxidation at 700 °C, thus supporting the μ-LEED measurements presented in Figure 6g–i, pointing to a connection between structural order and redox activity.

This hypothesis has been tested on ceria grown at 890 °C, yielding completely amorphous CeO_*x*_ nanoparticles previously discussed. The corresponding XAS spectra of the Ce M_5_-edge following different treatments are shown in Figure 7b, showing that the ceria is virtually reduced to Ce_2_O_3_ after deposition and does not show any significant changes during reduction and reoxidation attempts, corroborating the proposed structure–activity relationship; i.e., the formation of amorphous cerium oxide may have considerable influence on the redox activity of the ceria nanoparticles, although other factors such as initial deposit stoichiometry cannot be dismissed completely.

**Figure 8:**
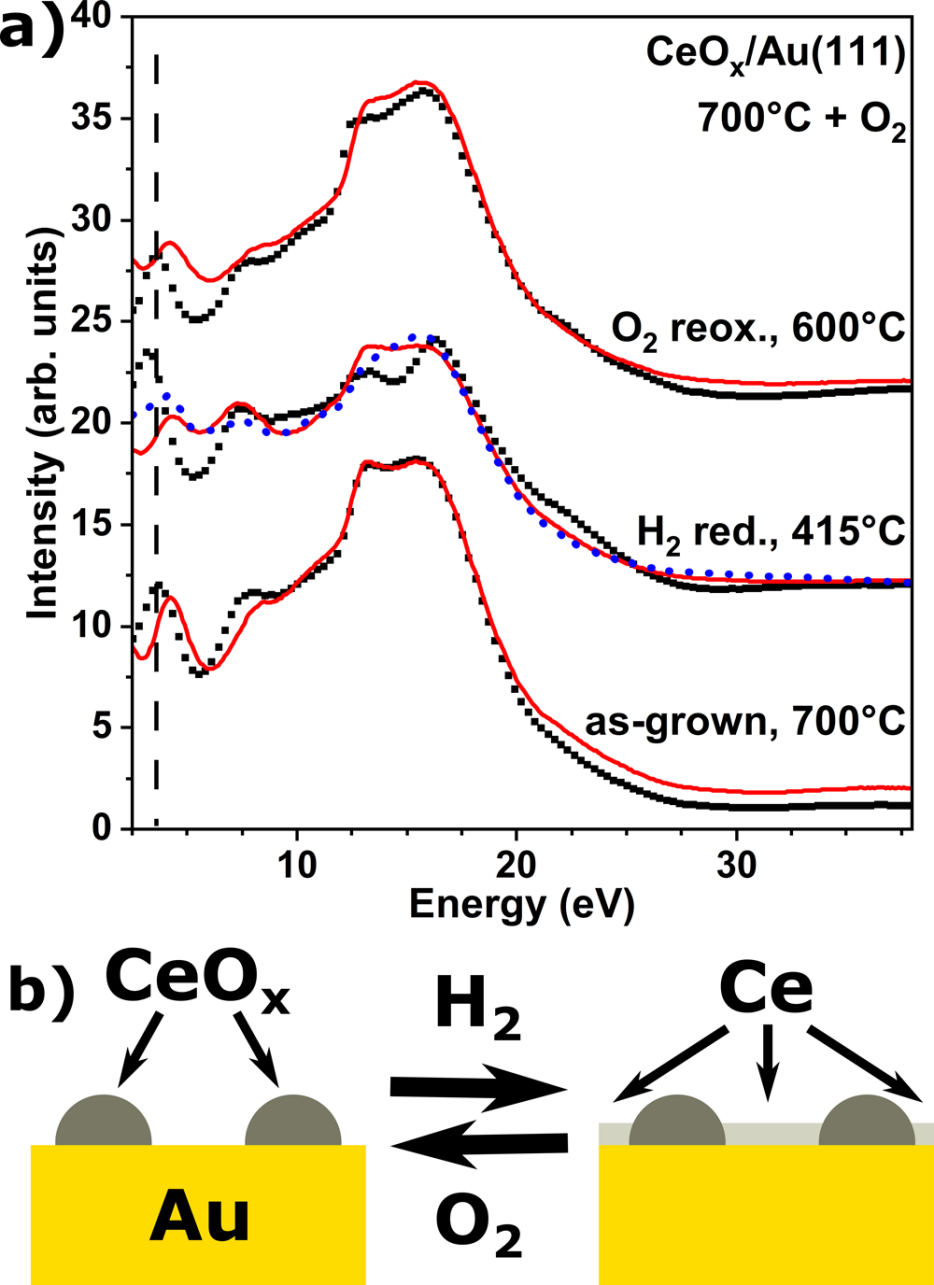
(a) I(V)–LEEM curves of the same sample as-grown, after H_2_ reduction at 415 °C, and O_2_ reoxidation at 600 °C (black dots) and fits using reference curves without metal (red solid lines) and with metal contribution (red dashed line); (b) sketch of a proposed surface model of changes induced during reduction and oxidation.

To unravel in more detail the changes observed during the reduction (H_2_) and reoxidation (using O_2_ or CO_2_) steps at mild temperatures (415 and 600 °C, respectively), the intensity evolution of the μ-LEED spots associated with ceria and gold has been monitored, as well as the I(V)–LEEM curves of CeO_*x*_/Au(111) grown at 700 °C in O_2_ after different treatments (Figure 8a). As mentioned previously, the ceria spots show an increase in intensity during exposure to H_2_, with a concomitant decrease in the intensity of the first-order gold spots, which would be consistent with a partial covering of the pristine Au(111) surface due to the decomposition of cerium oxide nanoparticles to cerium metal in the reducing ambient environment, which may form a wetting process. Due to the absence of the (√3 × √3) superstructure expected for the CeAu_2_ alloy,^24^ the formation of this Ce–Au alloy is not observed. However, the presence of other, possibly disordered Ce–Au alloys might serve as an alternative explanation regarding the presence of the Ce metal proposed here. Following our hypothesis, the lifting of the surface reconstruction would then result from the strong interaction between the cerium metal and gold. Further support for this hypothesis could be obtained from analyzing the I(V)–LEEM curves recorded before and after reduction (Figure 8a). Indeed, a significant redistribution of intensity can be observed, as expected on comparison of the reference curves of CeO_2_ and Ce_2_O_3_ (shown in Figure 2a). Interestingly, a slight change of the peak position around electron energies of 3–4 eV is visible (a vertical dashed line is added to guide the reader’s eye), which cannot be simply explained by a partial or total conversion from Ce^4+^ to Ce^3+^; a possible explanation is the appearance of cerium metal, which would correspond to a first peak at lower energies than what is observed for CeO_2_. Based on these observations, the I(V) curves in Figure 8a have been fitted by a linear combination of reference curves for clean Au(111), CeO_2_(111), and Ce_2_O_3_(111) (red solid lines), with the added possibility (red dashed line in Figure 8a) of including a fourth I(V)–LEEM curve of Ce metal [from Ce/Au(111), not shown here]. This approach yields fully oxidized CeO_2_ for as-grown CeO_*x*_/Au(111) prepared at 700 °C with O_2_, reducing to CeO_1.75_ and CeO_1.66_ with and without metal, respectively. Although the fits differ from the measurements in the low-energy range, the fit including the metal contribution reproduces the observed movement of the first peak to lower energies. Moreover, the Ce metal would form small ceria nanoparticles during the reoxidation step, dewetting the gold substrate and leading to the reappearance of herringbone reconstruction. This scenario would also explain the drop in the intensity of the ceria reflections in the μ-LEED patterns and the intensity increase of the Au beams, thus qualitatively reproducing the intensity evolution observed for the reoxidation. We note that, as Ce^3+^ and Ce^0^ XAS Ce M_4,5_ spectra are indistinguishable, the presence of an ultrathin layer of reduced cerium oxide interacting with the metallic substrate and presenting a distinct IV-curve could lead to similar results as considering a pure metallic Ce layer. I(V)–LEEM after reoxidation with O_2_ at 600 °C restores the features of the as-grown sample almost completely, indicating fully reversible redox behavior under these conditions.

Comparing these hints for the appearance of metallic cerium during the reduction to the literature on CeO_2_ growth on transition metal surfaces reveals similarities between the observations outlined by Höcker et al. for CeO_2_(111)/Ru(0001).^39^ In that study, the reduction to Ce_2_O_3_ followed by partial decomposition of the cerium oxide islands was reported after continued thermal annealing at 700 °C in UHV for about 1 h. The explanation given by the authors implies that the strong interaction between ceria and ruthenium stabilizes the oxide for long periods under aggressive reduction conditions until an increased number of oxygen vacancies in Ce_2_O_3_ favors the appearance of cerium metal. A similar phenomenon could be responsible for the observed changes to the Au(111) surface and the loss of the characteristic reconstruction, possibly enabled by the strong interaction between Ce metal and Au compared to CeO_*x*_ and Au.^24^ Compared to the Ru(0001) substrate, the weak interaction between Au(111) and CeO_*x*_ would lead to earlier oxide decomposition, especially considering the comparatively strong interaction with metallic Ce. Besides, this could also explain the enhanced growth stability using O/O_2_, as atomic oxygen might promote a stronger interaction between gold and the oxide islands during the nucleation process.

**Figure 9:**
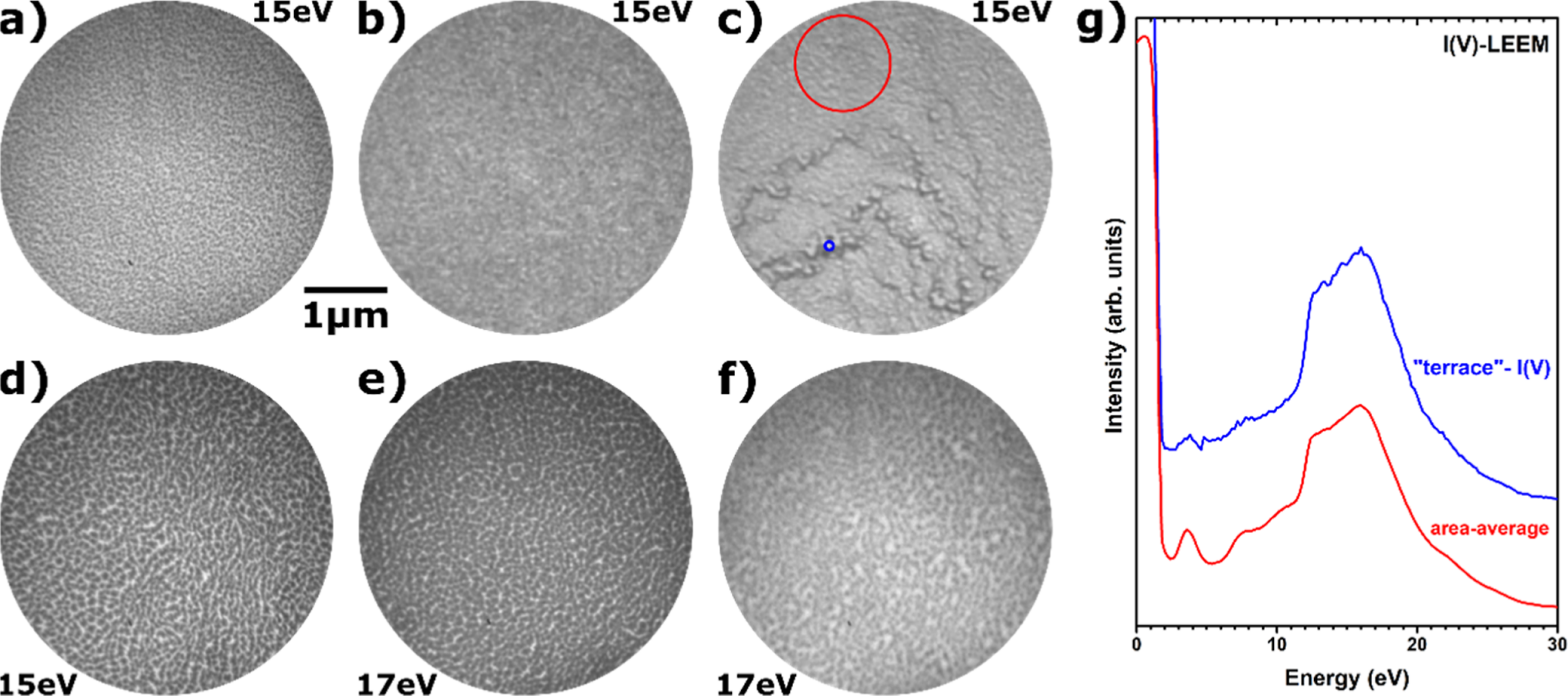
LEEM images of (a) as-grown CeO_*x*_/Au(111) prepared at 700 °C in O_2_, (b) after H_2_ reduction at 415 °C, and (c) after O_2_ reoxidation at 600 °C; (d) as-grown CeO_*x*_/Au(111) prepared at 750 °C using O/O_2_, (e) after H_2_ reduction at 415 °C, and (f) after CO_2_ reoxidation at 600 °C; (g) local I(V)-LEEM curves extracted from the areas indicated in (c).

With the proposed surface model, shown as a simple sketch in Figure 8b, some visible surface changes would be expected to appear in LEEM images, as shown elsewhere.^39^ Unfortunately, the small size of the oxide particles complicates the direct observation of this phenomenon. As displayed in the sequences Figure 9a–f for CeO_*x*_/Au(111) grown at 700 and 750 °C using O_2_ and O/O_2_, respectively, the surface does undergo some subtle changes. After H_2_ reduction, less of the bright gold substrate seems to be visible (compare Figure 9a to Figure 9b and Figure 9d to Figure 9e, respectively), concomitant with a decrease in the contrast between oxide nanoparticles and the substrate. Contrarily, reoxidation leads to the emergence of larger bright patches (compare Figures 9c to 9b and 9a and Figures 9f to 9e and 9d, respectively). For the sample grown at 700 °C in O_2_, local I(V)–LEEM curves extracted from the blue circle in Figure 9c (blue line in Figure 9g) show a strongly diminished ceria contribution when compared to an area-averaged I(V)-curve (red line in Figure 9g, extracted from the red circle in Figure 9c). However, this is not observed for reoxidized CeO_*x*_/Au(111) deposited at 750 °C in O/O_2_, showing a rather homogeneous distribution of the ceria signal in I(V)–LEEM curves and no ceria-free areas.

## 4 Discussion

The previous section presented a comprehensive overview of the CeO_*x*_/Au(111) growth characteristics at substrate temperatures between 700 and 890 °C and the influence of the oxygen chemical potential. In addition, the redox properties of selected systems were investigated under reducing (H_2_ ambient) and oxidizing (O_2_ or CO_2_) conditions.

Notably, the investigation of ceria growth using an O/O_2_ mixture provided by an atomic oxygen source shows a distinct influence of the oxygen chemical potential on the structural and chemical properties of the as-grown ceria nanoparticles, providing better-ordered deposits at higher temperatures than when using molecular oxygen. This increase in the structural order is likely due to a stronger interaction of the atomic oxygen from the O/O_2_ mixture with the Au(111) surface following a mechanism that seems to enhance the nucleation and adhesion of small ceria clusters. Nevertheless, the low affinity between oxygen and the inert Au(111) surface limits the effect when trying to vary the oxygen potential in terms of initial oxygen adsorption, especially when compared to other metallic substrates, such as Cu(111).

As briefly introduced in the previous section, a possible factor in explaining the observed nonmonotonic relationship between island size and deposition temperature and the generally small island size compared to other substrates might be the particularly weak interaction between Au and oxygen. As shown for other substrates,^30,41,46^ the presence of adsorbed oxygen can be identified by a characteristic superstructure observed in μLEED patterns, which may prime the surface and favor a layer-by-layer growth mode over island growth, yielding predominant growth in the lateral direction and, therefore, larger islands. In contrast, the low oxygen affinity of Au(111), indicated by the absence of an oxygen adsorbate layer in LEED, may drastically reduce the residence time and surface diffusion of cerium oxide precursor species that are increasingly reduced at higher temperatures, favoring the nucleation of small, reduced oxide nanoislands at adsorption sites provided by the herringbone reconstruction over attachment to existing islands.^19,25^ Combined with the changes imparted on the herringbone reconstruction at elevated temperatures, as reported by Abernathy and co-workers,^47^ and anisotropic diffusion on the surface due to the reconstruction, all these factors may constitute a potential explanation for the nonlinear evolution of island size with increasing substrate temperature.

This deficiency in initial adsorbed oxygen could also help to explain the as-grown Ce^3+^/Ce^4+^ mixtures reported on Au(111) at all substrate temperatures considered in this work. In addition, systems prepared in ambient O/O_2_ show superior stability at 415 °C under UHV conditions, potentially indicating a novel approach to preparing model catalysts composed of ceria nanoparticles on metal substrates with improved stability. Further investigation of the growth and nucleation dynamics of CeO_*x*_/Au(111) at different temperatures as well as ceria growth studies involving other substrates can help to deepen the understanding of the oxygen chemical potential on oxide stability and growth. Changes in the particle size distribution and their structural order caused by reactive oxygen might help to tune model catalyst properties toward improved activity and selectivity in catalytic applications.

The peculiar interaction proposed for CeO_*x*_/Au(111) under reducing and oxidizing conditions may help in understanding the nature of the metal–oxide interaction and the stability of model catalyst systems. Here, the proposed decomposition of ceria nanoparticles to cerium metal with concomitant wetting of the pristine Au(111) surface without sustained reduction to Ce_2_O_3_ can be understood from the strong interaction of Ce metal and Au compared to CeO_*x*_ and Au,^24^ although the presence of a disordered Ce–Au alloy instead of pure Ce metal cannot be ruled out completely. In comparison, the higher affinity of ruthenium toward oxygen might help stabilize ceria during the growth process, allowing for well-ordered growth even at high temperatures and enabling reduction to Ce_2_O_3_, with oxide decomposition only occurring after continued reduction, as described elsewhere.^39^ By performing similar studies on cerium oxide grown on other metal systems with varying affinity toward oxygen, e.g., Cu and Ag as other coinage metals with higher oxygen affinity than Au, valuable additional insights on the metal–oxide interaction might be gained to test if oxygen affinity constitutes a major component of this interaction. An improved understanding will help in the design of promising inverse oxide–metal model catalysts for catalytic applications.

## 5 Conclusions

We demonstrate a strong influence of substrate temperature and oxygen chemical potential on the structural and chemical properties of CeO_*x*_/Au(111) grown by cerium evaporation in molecular oxygen. At 700 °C substrate temperature, the as-grown particles are well-ordered and composed of fully oxidized CeO_2_ with a size of some 10 nm. With increasing temperature, the oxide particles exhibit progressively higher amounts of Ce^3+^ as derived from I(V)–LEEM and XAS measurements, culminating in fully reduced Ce_2_O_3_ at 890 °C. This trend is accompanied by a gradual loss of structural order, as inferred from microillumination low-energy electron diffraction. Notably, the size of the oxide islands shows a trend reversal with deposition temperature, increasing from 700 to 750 °C, followed by a distinct decrease in particle size at 890 °C. Using reactive atomic oxygen leads to well-ordered and more stable ceria nanoislands at 750 °C substrate temperature, contrary to the use of molecular oxygen under identical conditions. These changes induced by the higher oxygen potential on the Au(111) surface when using reactive oxygen are in good agreement with previous reports in the literature and highlight the strong influence of growth conditions on the catalytic behavior and the metal oxide/metallic support interaction. Notably, the oxygen affinity of the metallic substrate, and thus the possibility of adjusting the initial oxygen potential, seems to have a crucial influence on the growth of ceria in terms of initial stoichiometry, structural order, and epitaxial growth.

Furthermore, exposure to subsequent reducing and oxidizing atmospheres has shown a distinct disappearance of structural order during H_2_ reduction at high temperatures without reappearance during reoxidation attempts with O_2_ and no evident change in composition during reoxidation, implying a connection between reversible redox behavior and structural order. The lower interaction between the Au(111) surface and the CeO_*x*_ nanoislands than for metallic Ce seems to lead to the decomposition of the cerium oxide at more gentle conditions than when employing other metallic substrates, highlighting once again the complex synergistic interaction between the substrate and metal oxides under redox conditions. Thus, special care on growth conditions might open new routes for preparing new inverse model catalysts with improved temperature stability.
